# Union rates and functional outcome of double plating of the femur: systematic review of the literature

**DOI:** 10.1007/s00402-021-03767-6

**Published:** 2021-01-23

**Authors:** M. F. Lodde, M. J. Raschke, J. Stolberg-Stolberg, J. Everding, S. Rosslenbroich, J. C. Katthagen

**Affiliations:** Department for Trauma, Hand and Reconstructive Surgery, University Hospital Münster, Albert- Schweitzer-Campus 1, 48149 Münster, Germany

**Keywords:** Femoral fractures, Double plating, Non-union, Periprosthetic fracture, Polytrauma

## Abstract

**Background:**

The optimal treatment strategy for the surgical management of femur fractures and non-unions remains unknown. The aim of this study is to assess union rates, complications and outcome after femoral double plating. Treatment of shaft, distal, periprosthetic fractures and pathological proximal femur fractures as well as femoral non-unions with double plating were evaluated.

**Methods:**

A systematic review according to the Preferred Reporting Items for Systematic reviews and Meta-Analyses (PRISMA) statement was conducted. Published literature reporting on the treatment and clinical outcome of femoral fractures and non-unions with double plating was identified. In total, 24 studies with 436 cases of double plating, 64 cases of single plating, 84 cases of intramedullary nailing (IM), and 1 interfragmentary screw treatment met the inclusion criteria of this systematic review. The evaluated literature was published between 1991 and 2020.

**Results:**

Double plating of femoral fractures achieved high healing rates and few complications were reported. It displayed significantly less intraoperative haemorrhage, shorter surgery time reduced risk of malunion in polytraumatised patients when compared to IM. Fracture healing rate of double-plating distal femoral fractures was 88.0%. However, there were no significant differences regarding fracture healing, complication or functional outcome when compared to single plating. Treatment of periprosthetic fractures with double plating displayed high healing rates (88.5%). Double plating of non-unions achieved excellent osseous union rates (98.5%).

**Conclusions:**

The literature provides evidence for superior outcomes when using double plating in distal femoral fractures, periprosthetic fractures and femoral non-unions. Some evidence suggests that the use of double plating of femoral fractures in polytraumatised patients may be beneficial over other types of fracture fixation.

**Level of evidence:**

IV.

## Introduction

Double plating is performed by adding a second implant for fracture treatment increasing stability and providing advantages of fracture fixation in regions with high bending forces and intra-articular metaphyseal fractures [1]. Due to these biomechanical advantages, there are several indications for double plating of the femur including femoral fractures, pathological fractures of the proximal femur, periprosthetic femoral fractures and femoral non-unions [2–4].

In general, the treatment of femoral shaft fractures with intramedullary nailing (IM) or single-plate fixation can achieve high union rates [5]. However, some evidence in the literature shows a 24% rate of complications, including non-union, implant failure and infection [6]. Particularly in polytraumatised patients, plate fixation of the femoral shaft can provide a successful treatment option [7]. Plate fixation is recommended in patients with lung injuries unsuitable for IM [7, 8].

A further indication for double plating of the femur is distal femoral fractures. These injuries occur in young patients after high-energy or much more frequently in the older patients after low-energy trauma. Fractures of the distal femur account for 3–6% of all femoral fractures [9, 10]. Plating and IM are discussed in the literature as treatment options, with plating used predominating. Furthermore, biomechanical studies report better results with plating compared to other fixation methods [11]. A recent meta-analysis displayed a non-union rate of approximately 5% after fixation of distal femur fractures using a single locking compression plate or retrograde intramedullary nailing [12].

In addition, periprosthetic fractures of the femur can be treated successfully with double plating. Periprosthetic fractures following hip or knee arthroplasty are difficult to treat because they are associated with poor bone quality and distal fragments of insufficient length for adequate fixation [13].

Treatment options for pathological proximal femur fractures include intramedullary nailing (IM) [14], endoprosthetic reconstruction [15] and plating with and without augmentation of bone cement [16]. The compound osteosynthesis has been developed for these pathological fractures [17, 18]. The reconstruction of the proximal femur and fixation using a condylar plate together with an intramedullary placed narrow small fragment plate achieves a great amount of stability [17, 19] and is more stable compared to intramedullary nailing [18, 20, 21]. Furthermore, functional results of double plating are superior compared to primary endoprosthetic replacement [22].

Another indication for double plating of the femur is the treatment of non-unions. The overall rate of femoral non-union is approximately 14% [23]. Non-union of the femur can result from severe open fracture or segmental bone loss, infection or failure of the previous implants. Non-union in the lower extremities is associated with axial malalignment, loss of ambulatory function, decreased range of motion, chronic pain and reduced quality of life [24].

Given that fracture healing varies between diaphyseal and metaphyseal bone or pathological fractures and that accompanying injuries as well as comorbidities and injuries of the soft tissues between polytraumatised patients and the elderly patient suffering from periprosthetic fractures are different, the most important common denominator of this review are the surgical technique of double plating and the anatomical femoral region.

Endpoints of the present study were union rates and complication rates for double plating of femoral shaft fractures, distal femoral fractures, periprosthetic femoral fractures, pathological fractures of the proximal femur and femoral non-unions. When possible, comparison to other fixation procedures was to be performed. It was hypothesised that double plating of the femur for each indication mentioned above achieves high union rates with low complication rates and that double plating is a successful alternative compared to other fixation procedures.

## Materials and methods

**Fig. 1:**
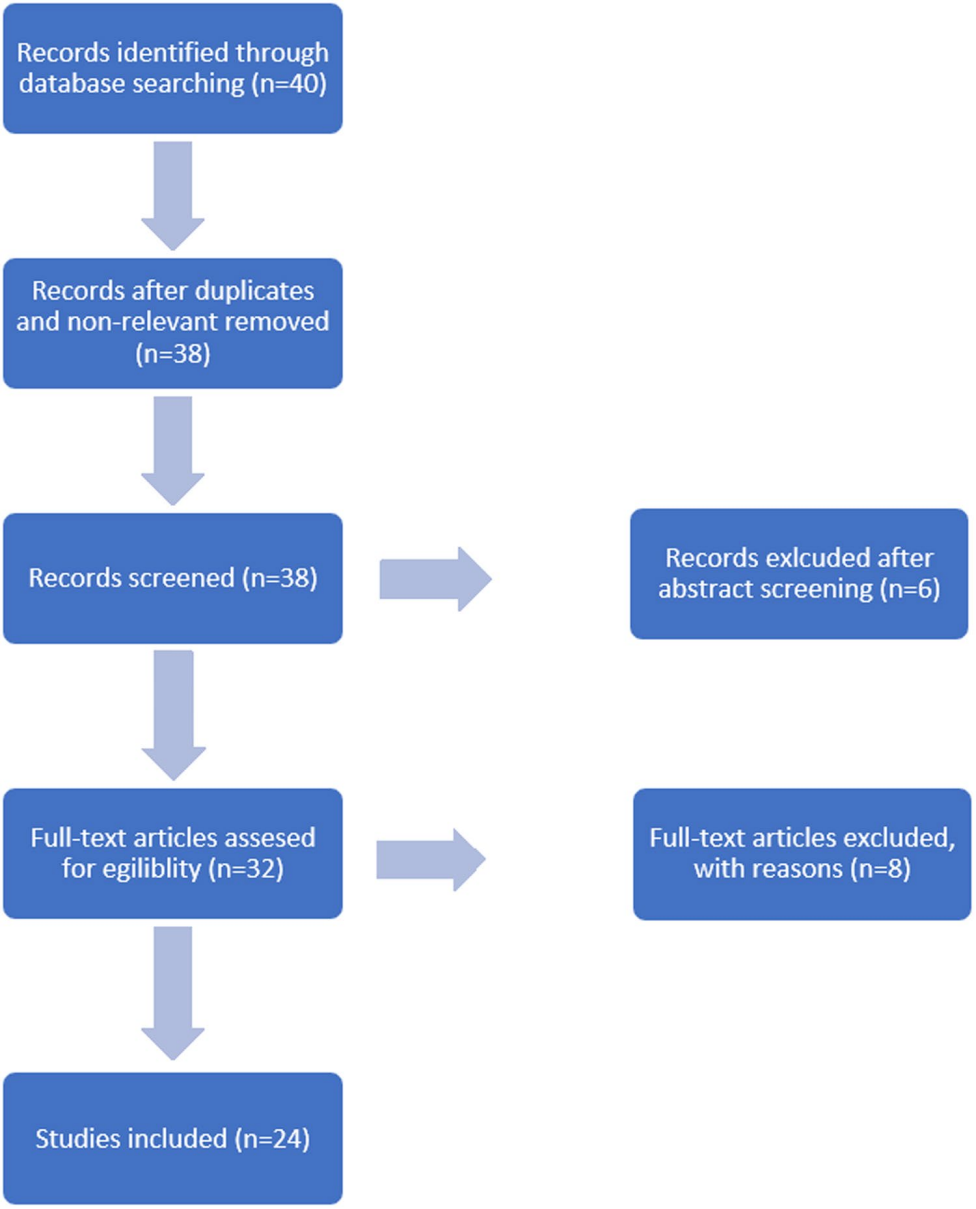
PRISMA flow chart showing the path from identification to inclusion of relevant literature

Electronic database was searched to identify all published literature addressing the treatment of fractures and non-unions of the femur. This study was conducted in accordance with the 2009 preferred reporting items for systematic review and meta-analysis (PRISMA) statement (Fig. 1) [25]. The search was performed using PubMed in November 2020. According to the predefined selection criteria, studies that had been published from the database inception until November 30, 2020 were searched using both MeSH (Medical Subject Headings) terms and keywords. The search terms were ”femoral fracture AND dual plating”, “femoral fracture AND double plating”, “fracture of the femur AND dual plating”, “fracture of the femur AND double plating”, “periprosthetic femoral fracture AND dual plating”, “periprosthetic femoral fracture AND double plating”, “periprosthetic fracture of the femur AND dual plating”, “periprosthetic fracture of the femur AND double plating”.

Two investigators independently reviewed the titles, abstracts and texts from all included articles. Selection for inclusion was determined by examining the title and abstract of all articles obtained from the search (Fig. 1). The citations in the included studies were manually checked to identify potentially eligible studies. Full-text articles were examined for review to allow further assessment of inclusion and exclusion criteria (Fig. 1).

A study was included when it (1) assessed the treatment of fractures or non-unions of the femur with two plates, with two plates compared to a single plate or compared to IM and (2) consisted of a cohort of seven or more patients. A study was excluded when it (1) consisted of a cohort of less than seven patients, (2) was basic science or editorial articles or surgical technique descriptions or (3) was an animal or biomechanical study. The present study was conducted with language limitation to English, French and German.

The retrieved studies were populated in Citavi (version 6 Swiss Academic Software and Citavi, Wädenswil, Switzerland,) and then exported to Microsoft software (Microsoft 365 Business Standard, Redmond, WA USA). Following the exclusion of duplicate results, the study selection was performed.

Two reviewers independently extracted the key information from the included studies comprising the names of the authors, year of publication, level of evidence, number of patients, treatment, mean follow-up, fracture healing, complications and functional outcome at final follow-up. Descriptive analysis was performed with Excel. The level of evidence was assigned according to the classification as specified by Wright et al. [26]. In addition, studies were evaluated according the Coleman methodology score (CMS) [27].

## Results

A total of 24 studies, published between 1991 and 2020, met the inclusion criteria of this systematic review. Studies were level III or IV according to Wright et al. [26].

### Femoral shaft fractures in polytraumatised patients

One study examined outcomes of double plating of femoral shaft fractures [8]. A second study compared outcomes of double plating versus IM [28]. In total, 141 patients and 141 femoral shaft fractures were included (Table 1). Double plating was performed in 57 cases and IM in 84 cases. The mean follow-up was 16.5 months. Fracture healing was achieved in 138 cases (97.9%). The mean healing time was 8.8 months. The study comparing IM and double plating reported significantly less intraoperative haemorrhage when double plating was used for fixation of femoral shaft fractures [28]. Furthermore, a significantly shorter surgery time and a significantly reduced risk for malunion was observed [28]. All 141 cases were polytraumatised patients [8] or patients with an Injury Severity Score (ISS) ≥ 18 [28]. The overall complication rate was 35% among the 141 patients. The complication rate of double plating was 28% (16 complications in 57 cases), while that of IM was 40% (34 complications in 84 cases) (Table 1). Pulmonary complications were observed in 26 cases of the IM group (31%) and in 10 cases of the double-plating group (18%). However, there were no significant differences between the IM and the double-plating groups regarding function scores, fracture union rate, overall pulmonary complication rate or in-hospitality mortality [28]. The mean Coleman methodology score was 48 (Table 2).

- *n* = 1 delayed union (0.7%)
- *n* = 1 superficial wound infection (0.7%)
- *n* = 36 pulmonary complications (25.5%) (*n* = 26 IM group, *n* = 10 double-plating group)
- *n* = 5 multiple organ failure (3.6%)
- *n* = 7 mortality during hospitalisation (5.0%).

**Table 1:** Results of the systematic review for double plating of femoral shaft fractures

Title	Authors	Journal	Year	Level of evidence	Number of cases	Treatment	Mean follow-up (months)	Fracture healing	Complications	Functional outcome at final follow-up
Double-plating fixation of comminuted femoral shaft fractures with concomitant thoracic trauma	Cheng et al.	Journal of International Medical Research	2018	IV	*n* = 15 Mean age: 38.7 years Range of age: 21–58 years	LCP anterior surface of femoral shaft LCP or distal femoral locking titanium plate lateral	14.2 (SD 12–24)	Union was achieved in 14 of 15 patients Mean time of healing: 9.2 months	*n* = 2 1 delayed union 1 superficial wound infection	Range of motion of knee flexion 116.4 (+-6,9) Mean Nonarthritic Hip Score and Lysholm Knee Score was 89 (+-7.8) points and 87 (+ -8.4 points)
Interlocking Intramedullary Nailing Versus Locked Dual-Plating Fixation for Femoral Shaft Fractures in Patients with Multiple Injuries: a retrospective comparative study	Cheng et al.	Journal of Investigative Surgery	2019	IV	*n* = 126 84 patients treated with intramedullary nailing Mean age: 36.2 Range of age: 19–67 42 patients treated with locked dual plating Mean age: 38.6 years Range of age: 21–69 years	Interlocking intramedullary nailing Locked dual plating (LCP)	Intramedullary nailing 16.4 Locked dual plating 18.9	Bony union was achieved in 98.4% after 32.5 weeks and 36.3 weeks in the intramedullary nailing and locked dual plating groups	*n* = 48 IMN group: 26 pulmonary complications 3 multiple organ failures 5 deaths caused by ARDS, pulmonary contusion, multiple organ failure, brain trauma LDP group: 10 pulmonary complications 2 multiple organ failures 2 deaths caused by multiple organ failure	Harris Hip Score (points) Interlocking intramedullary nailing: 86.1 ± 10.2 Locked dual plating: 88.3 ± 9.4 Lysholm Knee Score (points) Interlocking intramedullary nailing: 85.1 ± 16.2 Locked dual plating: 83.3 ± 14.4

**Table 2:** The Coleman methodology score for double plating of femoral shaft fractures

Methodology criterion (max score)	Mean score (SD)
Part A	
1. Study size	5 (5)
2. Mean follow-up (months)	2 (0)
3. N procedures	10 (0)
4. Type of study	0
5. Diagnostic certainty	5 (0)
6. Description of surgical procedure given	5 (0)
7. Description of surgical procedure given	10 (0)
Part B	
1. Outcome criteria	2 (0)
2. Outcome assessment	4 (0)
3. Outcome assessment	5 (0)
Coleman methodology score (CMS)	48 (5)

### Distal femoral fracture

Six studies described outcomes of case series with double plating of distal femoral fractures [2, 29–33]. A total of 156 distal femoral fractures (AO [Arbeitsgemeinschaft für Osteosynthesefragen] 33 A–B and 33-C2 and 33-C3 fractures) were included (Table 3). Of these 156 cases, 108 distal femoral fractures were treated with double plating which achieved healing in 95 cases (88.0%). Bai et al. (2018) compared lateral plating in 48 cases and double plating in 12 cases of distal femoral fractures. No significant differences in the mean operation time, intraoperative haemorrhage or fracture-healing time were observed. Good to excellent knee function 1 year postoperatively was found [29]. While fracture healing was achieved in all cases in the double-plating group, there was one non-union in the single-plating group [29]. Summarising all 108 cases, the overall complication rate was 33.3%. The mean Coleman methodology score was 41.5 (Table 4).

- *n* = 9 infection (8.3%)
- *n* = 10 non-union (9.3%)
- *n* = 2 mortality during hospitalisation (1.9%)
- *n* = 3 mild pain at the iliac grafting donor site (2.8%)
- *n* = 2 arthrolysis under general anaesthesia at 3 weeks after surgery because of a clear delay in rehabilitation response and fear of development of arthrofibrosis (1.9%)
- *n* = 2 delayed tibial tuberosity osteotomy healing for more than 12 weeks (1.9%)
- *n* = 2 delayed wound healing (1.9%)
- *n* = 1 deep-vein thrombosis (0.9%)
- *n* = 6 screw breakage or cut-out in one of the plate fixations with no loss of reduction (5.6%).

**Table 3:** Results of the systematic review for double plating of distal femoral fractures

Title	Authors	Journal	Year	Level of evidence	Number of cases	Treatment	Mean follow-up (months)	Fracture healing	Complications	Functional outcome at final follow-up
Double-plating of comminuted, unstable fractures of the distal part of the femur	Sanders et al.	The Journal of Bone and Joint Surgery	1991	IV	*n* = 9 Mean age: 39 years Range of age: 21–75 years	Lag screw and condylar buttress plate lateral, in the distal part cancellous bone screws, medial plate and bone graft	26 (SD 2–34)	All 9 fractures healed in an average time of 6.7 months (SD 5–9 months)	*n* = 0	5 good and 4 fair functional results were achieved according to methods of Sanders et al. Flexion of the knee: 3 patients had < 90°, 5 patients 90°-100°, 1 patient > 100°. 1 patient was always pain free and none patient had pain at rest
Lateral and anterior plating of intra-articular distal femoral fractures treated via an anterior approach	Ziran et al.	Internantional Orthopedics	2002	IV	*n* = 36 displaced distal femoral fractures Mean age: n.a Range of age: n.a	Anterior approach and double-plating lateral plate (condylar or blade) and anterior plate (reconstruction or 3.5 mm dynamic compression)	7 (SD 3–44) two patients died during hospitalization, one patient was lost to follow-up	Uneventful healing by 16 weeks occurred in 24 of 36 cases 3 non-unions	*n* = 8 2 died during hospitalization (non-related cause to surgery), 3 non-unions 1 superficial wound infection 2 infection (1 of which resulted in amputation below the knee)	Mean arc of motion was from 5° (5°–35°) − 100° (20°–130°) flexion
Highly unstable complex C3-type distal femur fracture: can double plating via a modified Olerud extensile approach be a standby solution?	Khalil Ael-S et al.	Journal of Orthopedics and Traumatology	2012	IV	*n* = 12 Mean age: 33.5 years Range of age: 22–44 years	Ipsilateral iliac bone was draped for autografting Lateral locked distal femur plate Contoured medial plate (reconstruction plate in eight cases, semitubular plate in four cases) Lateral distal femur locked plate and a medial contoured plate through a modified Olerud extensile approach	13.7 (SD 11–18)	All cases had radiological healing. Mean healing time 18.3 months (SD 12–28 weeks) with 4 cases having a delayed union (more than 24 weeks)	*n* = 15 4 delayed unions (more than 24 weeks) 3 cases (25%) had mild pain at the iliac grafting donor site 2 cases (16.7%) had manipulation under general anaesthesia after 3 weeks from surgery due to manifest delay in rehabilitation response 2 superficial infections (16.7%), 2 delayed wound healings (16.7%) 2 delayed tibial tuberosity osteotomy healings for more than 12 weeks (16.7%)	7 of 12 cases had excellent and good results (58.4%); 3 cases had fair results (25%); 2 cases had poor results (16.7%) according to methods of Sanders et al
Double plating of intra-articular multifragmentary C3-type distal femoral fractures through the anterior approach	Imam et al.	European Journal of Orthopaedic Surgery & Traumatology	2017	IV	*n* = 16 supracondylar Femoral fracture type C3 Mean age: 36 years Range of age: 18–59 years	Countersunk cancellous screws size 4 or 6.5 mm followed by applying a distal femoral locked plate sub-musculary on the lateral surface of the condyle; application of contoured medial plate (proximal tibia in ten cases, distal tibia in six cases); bone grafting from the iliac bone	11.5 (SD 6–24)	Complete radiological union was 6.0 ± 3.5 months (SD 3–14 months) with one case having a delayed union	*n* = 4 1 nonunion (6.25%); 2 infections (12.5%); 1 needed revision (6.25%)	Eleven had well-to-excellent functional outcome (68.75%) Poor outcome in two patients (12.5%) according to methods of Sanders et al
Comparison of Clinical Efficacy of Lateral and Lateral and Medial Double-plating Fixation of Distal Femoral Fractures	Bai et al.	Scientific Reports	2018	IV	*n* = 60 distal femoral fractures *n* = 48 treated with lateral plate (single plate) *n* = 12 treated with lateral plate and medial plate (double plating) Mean age: n.a Range of age: n.a	Single-plate group Double plate group: after setting the lateral plate, if varus stress was positive and lateral collateral ligament rupture excluded, medial double plating was used	Single plate group: 15.2 Double plate group: 18.5	Single-plate group: union was achieved in 47 cases (97,9%) Mean time of healing: 14.3 Double plate group: union was achieved in all 12 cases 100% Mean time of healing: 18 months	Single-plate group: *n* = 1 1 non-union (2,1%), retreated with autologous iliac bone graft and lateral anatomical plate fixation, which then healed Double-plate group: *n* = 0	Single-plate group: excellent and good results in 39 of 48 cases (81.3%); fair results in 7 cases (14.6%) and poor results in 2 cases (4.2%) according to Kolmert´s standard Double-plate group: excellent and good results in 9 of 12 cases (75%); fair results in 2 cases (16.7%); poor results in 1 case (8.3%) according to Kolmert´s standard
Single-Incision Double-Plating Approach in the Management of Isolated, Closed Osteoporotic Distal Femoral Fractures	Metwaly et al.	Geriatric Orthopaedic Surgery and Rehabilitation (GOS)	2018	III	*n* = 23 distal osteoporotic femoral Fractures (AO 33-A3, 33-C1-3) Mean age: 69.6 years Range of age: 61–80 years	Medial: antishear plate (locked L-plate or medial distal femoral osteotomy locked plate) Lateral: long-locked lateral distal femoral plate minimally invasive percutaneous plate osteosynthesis (MIPPO)	14.1 SD (12–36)	Union was achieved in 19 cases (82.6%) 4 (17.4%) cases needed autologous bone graft after 6 months Mean time of union: 9 months (SD 3–12 months)	*n* = 9 (39.1%) 2 superficial infections 1 deep-vein thrombosis 6 screw breakage or cut-out in one of the plate fixations no loss of reduction	The mean EQ-5D-5 L score was 83.8 (72–92) 100 means the best health the patient can get and 0 means the worst health knee range of motion was 3°-5° less when compared to the contralateral nonfractured side

**Table 4:** The Coleman methodology score for double plating of distal femoral fractures

Methodology criterion (max score)	Mean score (SD)
Part A	
1. Study size	2.5 (2.7)
2. Mean follow-up (months)	1.8 (1.7)
3. N procedures	9.5 (1.1)
4. Type of study	1.7 (3.7)
5. Diagnostic certainty	5 (0)
6. Description of surgical procedure given	10 (0)
7. Description of surgical procedure given	
Part B	
1. Outcome criteria	2 (0)
2. Outcome assessment	4 (0)
3. Outcome assessment	5 (0)
Coleman methodology score (CMS)	41.5

### Periprosthetic femoral fracture

Five studies reported outcomes of case series with double plating of periprosthetic femoral fractures. Periprosthetic fractures around total hip and knee arthroplasty and femoral shaft were included [3, 34–37]. A total of 106 patients (109 cases) with 68 periprosthetic and 38 femur fractures, including 2 non-unions and 1 open fracture, were analysed (Table 5). Of these 109 cases, 96 cases were treated with double plating. The fractures contained type A3 and C3 fractures according to AO/OTA-classification. Very low supracondylar fractures and periprosthetic fractures around total hip and total knee arthroplasty (Vancouver classification B1 and C, Su et al. [38] Su2 and Su3) were included. Müller et al. [3] evaluated double plating of ten periprosthetic fracture cases following hip or knee arthroplasty: six periprosthetic fractures after total hip arthroplasty, three periprosthetic fractures after total knee arthroplasty and one interprosthetic fracture after total hip and knee arthroplasty. Seven patients were included in the final follow-up. Fracture healing was achieved in all cases [3]. The mean follow-up was 34.5 months (Table 5). Two patients passed away because of non-surgery related events before the follow-up [3]. Park et al. [36] analysed periprosthetic fractures around knee arthroplasty (Su type 3). Of 21 patients, 20 achieved union at an average of 14 weeks postoperatively and the mean Knee Society knee and function scores were 94 and 89.5 [36]. Bologna et al. [37] showed in their retrospective study that double plating of complex distal femoral fractures (AO/OTA 33-C2/33-C3) or periprosthetic fractures led to significantly higher union rates compared to single plating. It is to highlight that of 13 cases treated with single plating only, there were 6 non-unions and 4 delayed unions (76.9% impaired bone healing).

**Table 5:** Results of the systematic review for double plating of periprosthetic fractures

Title	Authors	Journal	Year	Level of evidence	Number of cases	Treatment	Mean follow-up (months)	Fracture healing	Complications	Functional outcome at final follow-up
Clinical and radiological results of patients treated with orthogonal double plating for periprosthetic femoral fractures	Müller et al.	International Orthopaedics	2014	IV	*n* = 10 *n* = 5 fractures around a total hip prosthesis *n* = 1 fracture around a total knee prosthesis *n* = 1 interprosthetic fracture *n* = 3 implant failure after the stabilisation of periprosthetic fractures around a total hip prosthesis *n* = 1 or total knee prosthesis *n* = 2 mean age: 79.5 years range 55–91 years	Double plating lateral plating: 13–20 holes: LISS, LCP, NCB Anterior plating: 8–14 holes: LCP	22.6 SD [6–42] 2 patients lost due to dead not related to surgery	7 of 8 fractures healed within follow-up	*n* = 1 1 implant failure	7 patients presented with radiologically confirmed bony consolidation with the repaired extremity bearing their full weight All patients were subjectively satisfied with their clinical outcome Therefore, 7 out 10 patients showed excellent results according to Beals and Tower
A double-plating approach to distal femur fracture: A clinical study	Steinberg et al.	Injury	2017	IV	*n* = 32 *n* = 8 periprosthetic fractures *n* = 24 femoral fractures including 2 non-unions and 1 open fracture mean age: 76 years range of age: 44–101 years	Lateral locking plate and medial plate	12 SD [8–20]	30 of 32 fractures healed within 12 weeks (range 6–21 weeks)	*n* = 5 1 delayed union 1 a shaft fracture 2 superficial wound infections 1 deep infection after union	All fractures, excluding 1 that needed bone grafting and 1 refracture, healed radiographically within a mean of 12 weeks (range 6–21 weeks) and clinically within 11 weeks (range 6–17 weeks) Axial alignment was good in all cases, but 1 fracture had a valgus of 8°. Range of motion were for extension 0°-20° and for flexion 85°–120°
An alternative treatment for osteoporotic Su Type III periprosthetic supracondylar femur fractures: Double locking plate fixation	Çiçek et al.	Acta Orthopaedica et Traumatologica Turcica	2018	IV	*n* = 22 distal femur periprosthetic fractures following total knee arthroplasty Su Type 3 mean age: 73 years range of age: 68–82 years	Medial and lateral locking plate (LCP) *n* = 18 spongious autograft	69 SD[53–85]	20 of 22 fractures healed within 18.5 weeks (range 14.2–22.8 weeks)	*n* = 3 1 non-union 1 loss of reduction 1 superficial infection	Union was observed in 20 patients KSS was 81.8 ± 7.8 (range 56–90) WOMAC score was 78.1 ± 5.3 (range 62–88) ROM was 98.1° ± 8.2° (range 70°–110°) Mean time to pain-free weight bearing was 4.9 ± 1.1 (range 4–8) months
Dual plate fixation results in improved union rates in comminuted distal femur fractures compared to single plate fixation	Bologna et al.	Journal of Orthopaedics	2019	IV	*n* = 21 distal femoral fractures *n* = 13 treated with lateral distal femoral locking plate (single plate) *n* = 8 treated with precontoured distal femoral locking plate lateral and straight locking plate anteromedial (double plating) Mean age: 61 years Range of age: n.a	Standard lateral approach Lateral distal femoral locking compression plate (single plate) Extensile parapatellar approach (double plating) Precontoured distal femoral locking plate was placed along the lateral distal femur, a straight locking plate was placed to the anteromedial surface of the distal femur	12 [6–29]	Single plate: 4 of 13 healed Dual plate: 8 of 8 healed	Single plate: *n* = 10 6 non-unions 3 delayed unions 1 infection Dual plate: *n* = 4 2 significant knee stiffness 2 mild anterolateral heterotopic ossifications	Single plate: knee range of motion 100° (92.5–115º) Dual plate: knee range of motion 90° (70.0–90.0°)
Excellent outcomes after double-locked plating in very low periprosthetic distal femoral fractures	Park et al.	Archives of Orthopaedic and Trauma Surgery	2020	IV	*n* = 21 distal femur periprosthetic fractures following total knee arthroplasty Su Type 3 Mean age: 76 years range of age: 56–90 years	Lateral: LCP Medial: LCP and Philos to the distal femur	12	20 of 21 fractures healed within 14 weeks (range 10–21 weeks)	*n* = 8 1 non-union 3 superficial infection 4 soft tissue healing	Of the 21 fractures, 20 (95%) healed primarily within 14 weeks (range 10–21 weeks). All cases achieved satisfactory limb alignment, with an m-LDFA of 89.6º (range 85–92°) and m-PDFA of 86.5º (range 70–130°) on average Average ROM: 114° (range 70–130°) The mean Knee Society knee and function scores were 94 (range 83–100) and 89.5 (range 76–99), respectively

Overall, healing was achieved in 85 of 96 cases fractures treated with double plating (88.5%) [3, 34–37]. The complication rate was 21.9%. The mean Coleman methodology score was 46.2 (Table 6).

- *n* = 1 non-union (1.0%)
- *n* = 2 implant failure (2.1%)
- *n* = 3 delayed union (3.1%)
- *n* = 6 superficial wound infection (6.3%)
- *n* = 1 reduction loss (1.0%)
- *n* = 1 deep infection (1.0%)
- *n* = 4 complication in soft tissue healing (4.2%)
- *n* = 2 significant knee stiffness (2.1%)
- *n* = 2 mild anterolateral heterotopic ossification (2.1%)

**Table 6:** The Coleman methodology score for double plating of periprosthetic fractures

Methodology criterion (max score)	Mean score (SD)
Part A	
1. Study size	3.2 (1.6)
2. Mean follow-up (months)	2.6 (1.2)
3. N procedures	9.4 (1.2)
4. Type of study	0 (0)
5. Diagnostic certainty	5 (0)
6. Description of surgical procedure given	5 (0)
7. Description of surgical procedure given	10 (0)
Part B	
1. Outcome criteria	2 (0)
2. Outcome assessment	4 (0)
3. Outcome assessment	5 (0)
Coleman methodology score (CMS)	46.2

### Pathological fractures of the proximal femur

Two studies analysed double-plate compound osteosynthesis for treatment of pathological fractures of the proximal femur (Table 7) [16, 18]. Merckaert et al. [18] reported that double-plate compound osteosynthesis is superior compared to other fixation techniques. Comparing the double-plate compound osteosynthesis and the single-plate compound osteosynthesis Kinkel et al. [16] showed that the double-plate technique is more stable and associated with a higher survival probability after 5 years. The mean Coleman methodology score was 49 (Table 8).

**Table 7:** Results of the systematic review for double plating of pathological fractures of the proximal femur

Title	Authors	Journal	Year	Level of evidence	Number of cases	Treatment	Mean follow-up (months)	Outcome	Functional outcome
Double-plate compound osteosynthesis for pathological fractures of the proximal femur: high survivorship and low complication rate	Merckaert et al.	Archives of Orthopaedic and Trauma Surgery	2019	IV	*n* = 61 Mean age: 63.5 Range of age: 39.6–92.7	*n* = 46 double-plate compound osteosynthesis *n* = 15a double-plate compound osteosynthesis was performed as revision procedure	22 (2.4–306)	Construct survival rates of 96% at 6 months, and 90% thereafter for primary reconstructions Comparing the calculated survivorship with the literature, it is evident that double-plate compound osteosynthesis is superior to simple open reduction and internal fixation with or without cement augmentation, intramedullary nailing and comparable if not higher than endoprosthetic replacement	The mean Merle d’Aubigné score was 14 ± 7 (range 3–17), at 0–3 months, 13 ± 3 (7–18), at 3–6 months, 15 ± 3 (9–18), at 6–12 months and 15 ± 4 (8–18) thereafter
Compound osteosynthesis for osteolyses and pathological fractures of the proximal femur	Kinkel et al.		2009	IV	*n* = 34 n.a n.a	*n* = 34 *n* = 22 double-plate compound osteosyntheses *n* = 12 single-plate compound osteosyntheses	n.a	Survival time after compound osteosynthesis was 14.2 months (range 0–72 months) Double-plate compound osteosyntheses showed a lower mechanical failure rate than single-plate compound osteosyntheses (14.3% vs 33.3%) and a higher survival probability after 5 years (76.4% vs 38.6%) No surgical revision was required due to perioperative complications in any case	n.a

**Table 8:** The Coleman methodology score for double plating of pathological fractures of the proximal femur

Methodology criterion (max score)	Mean score (SD)
Part A	
1. Study size	7 (3)
2. Mean follow-up (months)	1 (1)
3. N procedures	10 (0)
4. Type of study	0
5. Diagnostic certainty	5 (0)
6. Description of surgical procedure given	5 (0)
7. Description of surgical procedure given	10 (0)
Part B	
1. Outcome criteria	2 (0)
2. Outcome assessment	4 (0)
3. Outcome assessment	5 (0)
Coleman methodology score (CMS)	49

### Non-union of the femur

Nine studies reported outcomes of case series with double plating of femoral non-unions including the proximal femur, femoral shaft and distal femur [4, 24, 39–45]. Pydisetty et al. [42] analysed 10 patients with revision surgery for non-union of bisphosphonate-related subtrochanteric fractures. After removal of failed implants and resection of the non-union, bone grafting and double plating with a lateral dynamic compression screw (DCS) plate and anterior locking compression plate were performed achieving bone union in all the cases. However, one patient was lost to follow-up and complication rate was high in these complex fracture patterns [42]. Both, Lu et al*.* [43] and Mardani-Kivi et al*.* [44] reported that double plating and bone grafting achieved a healing rate of 100% for the treatment of atrophic distal femur non-union with bone defect and non-union of femoral supracondylar, subtrochanteric, and shaft fractures, respectively. There were no differences between double plating, single plate and interfragmentary screw in the only study comparing different fixation techniques [39]. A total of 193 non-unions were included (Table 9). Fracture healing was achieved in 190 cases (98.5%). Reported postoperative complications occurred with an overall rate of 25.9%. The mean Coleman methodology score was 44.2 (Table 10).

- *n* = 4 infection (2.1%)
- *n* = 1 sacral sore (0.5%)
- *n* = 2 postoperative seroma (1.0%)
- *n* = 4 blood transfusion (2.1%)
- *n* = 1 HDU admission (0.5%)
- *n* = 1 periprosthetic fracture (0.5%)
- *n* = 2 fatigue failure (1.0%)
- *n* = 1 malunion (0.5%)
- *n* = 1 persistent non-union (0.5%)
- *n* = 1 loss of motion of one knee (0.5%)
- *n* = 12 movement limitation (6.2%)
- *n* = 9 muscle atrophy (4.7%)
- *n* = 2 failure of attachment of the greater trochanter (1.0%)
- *n* = 7 symptomatic hardware (3.6%)
- *n* = 1 breakdown of the posterior iliac crest harvest site (0.5%)
- *n* = 1 deep-vein thrombosis (0.5%)
- *n* = 1 pulmonary embolism (0.5%).

**Table 9:** Results of the systematic review for double plating of non-union of the femur

Title	Authors	Journal	Year	Level of vidence	Number of cases	Treatment	Mean follow-up (months)	Fracture healing	Complications	Functional outcome at final follow-up
Treatment of Supracondylar Nonunions of the Femur with Plate Fixation and Bone Graft	Chapman et al.	The Journal of Bone and Joint Surgery	1999	IV	*n* = 18 Mean age: 47 years Range of age: 25–81 years	13 double plates, 4 single plates, and 1 interfragmentary screw Autologous bone graft used in all cases Condylar buttress plate antero-medial 95-degree screw and side-plate lateral	26 [SD 6–120] 1 patient lost to follow-up	All 18 non-unions had healed Average time to healing: 8 months (SD 3–20 months)	*n* = 3 1 infection 1 loss of motion of the knee 1 malunion	Range of motion of the knee was 101°(10°–135°)
Reattachment of complex femoral greater trochanteric nonunions with dual locking plates	Laflamme et al.	The Journal of Arthroplasty	2012	IV	*n* = 15 Mean age: 68 years Range of age: 42–88 years	2 locking plates contoured on the anterolateral and posterolateral surface of the greater trochanter Anterolateral plate: locking screws in the proximal and distal fragment	53.1 [SD 26–88]	Trochanteric union was achieved in 13 out of 15 cases	*n* = 5 1 associated stem fatigue failure had revision hip arthroplasty 1 failed reattachment 3 hardware removal	Trochanteric union was achieved in 13 patients (87%) Average total hip score was 14.8 ± 2.2 (Merle d'Aubigné) and 77.6 (± 12.8) (Harris Hip score)
Treatment of the femoral shaft nonunion with double plate fixation and bone grafting: A case series of 14 patients	Maimaitiyiming et al.	Injury	2015	IV	*n* = 14 Mean age: 26 years Range of age 22–32 years	Double-plate fixation combined with bone grafting LCP was placed in the lateral proximal part of the femur LCP which would make a 90° angle to the first plate was placed in the anterior site of the femur	14.8 [SD 10–25]	Union was achieved in all the patients in a mean of 5.5 months	*n* = 0	Excellent results in all cases according to the Paley and Catagni criteria
Addition of a Medial Locking Plate to an In Situ Lateral Locking Plate Results in Healing of Distal Femoral Nonunions	Holzman et al.	Clinical Orthopedics and Related Research	2016	IV	*n* = 23 Mean age: 58 years Range of age: 35–83 years	16 aseptic non-unions: single-stage procedure, stable lateral plate and medial LCP with autogenous bone graft 7 non-unions with lateral plate failure: 1. new lateral plate and at least 2 months later a medial LCP with autogenous bone graft	18 [SD 6–94] 2 patients lost to follow-up	Union was achieved in 20 out of 21 cases within 12 months	*n* = 6 1 persistent nonunion and lateral broken plate 4 removal of symptomatic hardware 1 breakdown of the posterior iliac crest harvest site	Union was achieved in 20 out of 21 cases within 12 months
Double locking plate fixation for femoral shaft nonunion	Peng et al	European Journal of Orthopaedic Surgery & Traumatology	2016	IV	*n* = 33 Mean age: 46.9 years Range of age: 25–81 years	Double-locking plate fixation and autogenous cancellous bone graft	24.8 (SD 6–60)	All 21 femoral non-unions healed Union time was 5.3 months (range 4–7)	*n* = 0	100% union rate, physical function and bodily pain components of the SF-36 were 96 (range 90–99) and 94.2 (range 92–99)
Treatment of atrophic nonunion via autogenous ilium grafting assisted by vertical fixation of double plates: A case series of patients	Sun et al.	Clinical Research Report	2019	IV	*n* = 21 femoral non-unions Mean age: 42 years Range of age: 23–68 years	Double plating was performed using a locking compression plate and a reconstructive plate, cancellous bone granules were loaded into any bony defects	14.5 (8–28)	All femoral non-unions healed Average union times 8.2 (range 4–14)	*n* = 13 9 muscle atrophy 4 joint ankyloses	100% union rate, all patients achieved an excellent or good result for bone healing and function
Double-plate fixation together with bridging bone grafting in nonunion of femoral supracondylar, subtrochanteric, and shaft fractures is an effective technique	Mardani-Kivi et al.	MUSCULOSKELETAL SURGERY	2019	IV	*n* = 41 Mean age: 35 years Range of age: 18–71 years	Double-plate fixation and autogenous bridging bone grafting	37 (SD 18–63)	Full union was obtained in all patients Union time was 5 months (range 4–8)	*n* = 9 1 deep-vein thrombosis 1 pulmonary embolism 7 movement limitations	100% union rate, at the final follow-up, 3 patients had 10°–20° movement 1 patient had 10° movement limitations in hip flexion and extension 3 patients had 20°–30° and 10° movement limitations in knee flexion and extension, respectively
J-bone graft with double locking plate: a symphony of mechanics and biology for atrophic distal femoral non-union with bone defect	Lu et al.	Journal of Orthopaedic Surgery and Research	2020	IV	*n* = 18 Mean age: 47.7 years Range of age: 28–63 years	Atrophic distal femur non-union with bone defect were treated with a combination of J-shaped iliac crest bone graft combined with double plate	22.1 (SD 14–34)	All 18 patients achieved primary bone healing Healing time was 6.7 months (range 3–12)	*n* = 3 2 superficial infections 1 knee stiffness	100% union rate, the mean time to weight bearing walking was 5.5 months (range 3–12) The rate of “excellent” and “good” Lysholm Knee Scoring Scale scores improved from 0% before surgery to 94.44% at 3 months after surgery
Outcome of revision surgery for bisphosphonate related subtrochanteric fracture non-union following failed intramedullary nailing	Pydisetty et al.	Injury	2020	IV	*n* = 10 Mean age: 71.5 years Range of age: 57–89 years	Lateral dynamic compression screw (DCS) plate and an anterior locking compression plate (LCP)	n.a 1 patient lost to follow-up	All 10 subtrochanteric non-unions healed Union time was 16 months (range 7–32)	*n* = 11 1 superficial infection 1 sacral sore 2 postoperative seroma 4 blood transfusion 1 HDU admission 1 periprosthetic fracture 1 fatigue failure	100% union rate, the mean period until fully weight bearing for all patients was 8 months (range 4–17 months

**Table 10:** The Coleman methodology score for double plating of non-union of the femur

Methodology criterion (max score)	Mean score (SD)
Part A	
1. Study size	2.1 (2.5)
2. Mean follow-up (months)	3.3 (2.1)
3. N procedures	7.8 (4.2)
4. Type of study	0
5. Diagnostic certainty	5 (0)
6. Description of surgical procedure given	5 (0)
7. Description of surgical procedure given	10 (0)
Part B	
1. Outcome criteria	2 (0)
2. Outcome assessment	4 (0)
3. Outcome assessment	5 (0)
Coleman methodology score (CMS)	44.2

## Discussion

The most important observations of this systematic review were (1) that double plating displayed significantly less intraoperative haemorrhage, a significantly shorter surgery time and a significantly reduced risk for malunion compared to IM in polytraumatised patients and thus it is reported to be a successful alternative to nailing, (2) that double plating of distal femoral fractures achieved very high healing rates (88.0%) with a reported overall complication rate (33.3%), (3) that double plating of periprosthetic femoral fractures displayed very high healing rates (88.5%) with a moderate overall complication rate of 21.9% and (4) that the treatment of femoral non-union with double plating achieved excellent osseous union rates (98.5%) with a reported overall complication rate of 25.9%.

Application of an external fixator in femoral fractures showed non-union rates of 0–12% [46]. Plate fixation of femoral fractures displayed non-union rates ranging from 1.6 to 8% [47–49]. The results of the present systematic review showed a 97.9% fracture-healing rate after double plating of femoral shaft fractures in polytraumatised patients. Moreover, better surgical parameters were observed. These findings might lead to an increase in the clinical use of double plating for the treatment of femoral shaft fractures in polytraumatised patients. Further studies are necessary to evaluate the possible benefits. According to current literature, treating femoral shaft fractures in polytraumatised patients with double plating is superior to IM.

Distal femur fractures occur in older patients after low-energy trauma and result from high-energy trauma in younger patients causing comminution, unstable fractures and bone loss [31]. Due to poor bone quality, treatment of distal femur fractures in older patients is difficult. Limitations of treatment with the condylar blade plate and supracondylar nailing are the reduction of the articular surface and fixation [31]. Furthermore, there is a high incidence of loss of fixation and varus collapse [2, 10]. Double plating of distal shaft fractures in other anatomical regions like the distal humerus is already a standard procedure [1]. According to biomechanical studies, a parallel arrangement of the plates appears to be best, however, there is no evidence for the optimal arrangement in clinical data [1]. In the examined case series, fracture healing was achieved in almost all patients treated with double plating, which might be advantageous over other fixation types. Compared to the results of the meta-analysis of Yoon et al. [12] showing a non-union rate of 5% after single-plate fixation or retrograde intramedullary nailing the results of the present study provide that double plating is a surgical treatment option. This systematic review shows that there is a lack of clinical studies comparing single versus double plating and other fixation techniques. According to the current literature examining double plating of the lower extremity, further clinical studies examining the best treatment options for the elderly patients are necessary.

Surgical treatment of periprosthetic fractures of the femur is challenging. Current literature reports high union rates (88.5%) of double plating of distal femoral and periprosthetic fractures following hip or knee arthroplasty. Considering the high mean age of 76.2 years of the patients, double plating is a valid treatment option. The present study reveals that double plating of periprosthetic fractures leads to a moderate rate of complications [3, 34]. Furthermore, double locking plating of osteoporotic periprosthetic supracondylar femur fractures resulted in reduction of complication rates, reduction loss and implant failure and allows early mobilisation and rehabilitation and earlier weight bearing [35]. However, currently there are predominantly level IV studies in the literature. One retrospective level III study showed significant better results for double plating compared to single plating [37]. In comparison to other fixation techniques, more clinical data are necessary to further evaluate the benefit of double plating of periprosthetic fractures. The potential advantages of full weight bearing after double plating and thus preventing complications, including pneumonia and thrombosis, needs further evaluation.

It is reported that using the double-plate compound osteosynthesis superior biomechanical characteristics and a higher survival probability can be achieved. However, due to a lack of consistency of reporting in the literature, more clinical data are necessary to underline the possible advantages of double plating for these fracture patterns.

Non-unions of the femur occur after open fractures and metaphyseal comminution [50]. The present systematic review shows union rates of 98.5% using double plating and bone grafting for the treatment of femoral shaft non-unions. Therefore, double plating for the treatment of femoral shaft union is a viable treatment option.

Supracondylar non-unions are serious complications and there is no treatment standard [39, 51]. The present study shows that a high proportion of patients with distal femoral non-unions can be treated successfully with double plating and autogenous bone grafting. Moreover, compared to other studies, the use of dual plating and bone grafting for the treatment of supracondylar femoral non-unions can achieve higher healing rates [24, 39, 52].

Stabilisation of the greater trochanter in the revision of total hip arthroplasty is a major challenge. Non-union rates using multibraided metallic cables range from 20 to 31% [40, 53, 54]. Operative techniques for the reattachment of the greater trochanter are cable fixation, single-plate devices and dual plating [40]. Current literature shows that double plating leads to considerably higher union rates than other fixation techniques [55–57]. According to current literature, a 100% union rate can be achieved when the trochanter is attached to the femur with good contact and double plating [40]. Superior results when performing double plating of non-union of the greater trochanter need to be confirmed. Current literature shows that double plating might be superior.

A limitation of the present study is that the included studies—with one exception being level III evidence based—are level IV evidence based and that the overall mean Coleman methodology score was only 45.8. Major areas of methodological deficiencies were study size and type of study [27]. There is a need for more prospective clinical studies comparing double plating to other fixation techniques with greater study sizes. Whereas there was consistency in the literature regarding the definition of bone union, the reported complication rates vary in part substantially due to the different study designs.

Regarding the endpoints of the present study double plating of femoral shaft fractures, distal femoral fractures, periprosthetic femoral fractures, pathological fractures of the proximal femur and femoral non-unions achieves high union rates with moderate complication rates. The hypothesis that double plating of femoral fractures and non-unions can achieve high union rates with low complication rates is confirmed. The hypothesis that double plating of femoral fractures and non-unions can provide a successful alternative to other fixation procedures is also supported by the findings of the present systematic review.

## Conclusions

The current literature contains evidence for high healing rates and superior outcomes when using double plating in distal femoral fractures, periprosthetic fractures and femoral non-unions. Some evidence suggests that the use of double plating of femoral fractures in polytraumatised patients may be beneficial over other types of fracture fixation.

## References

[1] Katthagen JC, Schliemann B, Michel PA, Heilmann LF, Dyrna F, Raschke MJ (2020). Clinical application and outcomes of upper extremity double plating. Zeitschrift fur Orthopadie und Unfallchirurgie.

[2] Sanders R, Swiontkowski M, Rosen H, Helfet D (1991). Double-plating of comminuted, unstable fractures of the distal part of the femur. J Bone Jt Surg Am Vol.

[3] Müller FJ, Galler M, Füchtmeier B (2014). Clinical and radiological results of patients treated with orthogonal double plating for periprosthetic femoral fractures. Int Orthop.

[4] Maimaitiyiming A, Amat A, Rehei A, Tusongjiang M, Li C (2015). Treatment of the femoral shaft nonunion with double plate fixation and bone grafting: a case series of 14 patients. Injury.

[5] Winquist RA, Hansen ST, Clawson DK (1984). Closed intramedullary nailing of femoral fractures. A report of five hundred and twenty cases. J Bone Jt Surg Am Vol.

[6] Loomer RL, Meek R, de Sommer F (1980). Plating of femoral shaft fractures: the vancouver experience. J Trauma.

[7] Apivatthakakul T, Chiewcharntanakit S (2009). Minimally invasive plate osteosynthesis (MIPO) in the treatment of the femoral shaft fracture where intramedullary nailing is not indicated. Int Orthop.

[8] Cheng T, Xia R, Yan X, Luo C (2018). Double-plating fixation of comminuted femoral shaft fractures with concomitant thoracic trauma. J Int Med Res.

[9] Court-Brown CM, Caesar B (2006). Epidemiology of adult fractures: a review. Injury.

[10] Gwathmey FW, Jones-Quaidoo SM, Kahler D, Hurwitz S, Cui Q (2010). Distal femoral fractures: current concepts. J Am Acad Orthop Surg.

[11] Ehlinger M, Ducrot G, Adam P, Bonnomet F (2013). Distal femur fractures. Surgical techniques and a review of the literature. Orthop Traumatol Surg Res OTSR.

[12] Yoon B-H, Park IK, Kim Y, Oh H-K, Choo SK, Sung Y-B (2020) Incidence of nonunion after surgery of distal femoral fractures using contemporary fixation device: a meta-analysis. Arch Orthop Trauma Surg 10.1007/s00402-020-03463-x 32388648

[13] Meneghini RM, Keyes BJ, Reddy KK, Maar DC (2014). Modern retrograde intramedullary nails versus periarticular locked plates for supracondylar femur fractures after total knee arthroplasty. J Arthroplast.

[14] Tanaka T, Imanishi J, Charoenlap C, Choong PFM (2016). Intramedullary nailing has sufficient durability for metastatic femoral fractures. World J Surg Oncol.

[15] Lane JM, Sculco TP, Zolan S (1980). Treatment of pathological fractures of the hip by endoprosthetic replacement. J Bone Jt Surg Am Vol.

[16] Kinkel S, Stecher J, Gotterbarm T, Bruckner T, Holz U (2009). Compound osteosynthesis for osteolyses and pathological fractures of the proximal femur. Orthopedics.

[17] Friedl W (1992). Die Doppelplattenverbundosteosynthese. Ein Verfahren zur primär belastungsstabilen Versorgung von Problemverletzungen des subtrochanteren bis suprakondylären Femurbereiches. Aktuelle Traumatologie.

[18] Merckaert SR, Fontanellaz-Castiglione CD, Fornari ED, Tannast M (2020). Double-plate compound osteosynthesis for pathological fractures of the proximal femur: high survivorship and low complication rate. Arch Orthop Trauma Surg.

[19] Ganz R, Isler B, Mast J (1984). Internal fixation technique in pathological fractures of the extremities. Arch Orthop Trauma Surg.

[20] Friedl W, Ruf W, Mischkowsky T (1986). Die Doppelplattenverbundosteosynthese bei subtrochanteren pathologischen Frakturen. Eine klinische und experimentelle Untersuchung. Der Chirurg; Zeitschrift fur alle Gebiete der operativen Medizen..

[21] Schöttle H, Sauer HD, Jungbluth KH (1977). Stabilitätsmessungen bei Osteosynthesen am proximalen Femur. Archiv fur orthopadische und Unfall-Chirurgie.

[22] Rompe JD, Eysel P, Hopf C, Heine J (1994). Metastatic instability at the proximal end of the femur. Comparison of endoprosthetic replacement and plate osteosynthesis. Arch Orthop Trauma Surg.

[23] Zura R, Xiong Z, Einhorn T, Watson JT, Ostrum RF, Prayson MJ, Della Rocca GJ, Mehta S, McKinley T, Wang Z (2016). Epidemiology of fracture nonunion in 18 human bones. JAMA Surg.

[24] Holzman MA, Hanus BD, Munz JW, O'Connor DP, Brinker MR (2016). Addition of a medial locking plate to an in situ lateral locking plate results in healing of distal femoral nonunions. Clin Orthop Relat Res.

[25] Moher D, Liberati A, Tetzlaff J, Altman DG (2010). Preferred reporting items for systematic reviews and meta-analyses: the PRISMA statement. Int J Surg (Lond, Engl).

[26] Wright JG, Swiontkowski MF, Heckman JD (2003). Introducing levels of evidence to the journal. J Bone Jt Surg Am.

[27] Coleman BD, Khan KM, Maffulli N, Cook JL, Wark JD (2000). Studies of surgical outcome after patellar tendinopathy: clinical significance of methodological deficiencies and guidelines for future studies. Victorian Institute of Sport Tendon Study Group. Scand J Med Sci Sports.

[28] Cheng T, Xia R-G, Dong S-K, Yan X-Y, Luo C-F (2019). Interlocking intramedullary nailing versus locked dual-plating fixation for femoral shaft fractures in patients with multiple injuries: a retrospective comparative study. J Invest Surg Off J Acad Surg Res.

[29] Bai Z, Gao S, Hu Z, Liang A (2018). Comparison of clinical efficacy of lateral and lateral and medial double-plating fixation of distal femoral fractures. Sci Rep.

[30] Imam MA, Torieh A, Matthana A (2018). Double plating of intra-articular multifragmentary C3-type distal femoral fractures through the anterior approach. Eur J Orthop Surg Traumatol Orthop Traumatol.

[31] Khalil AE-S, Ayoub MA (2012). Highly unstable complex C3-type distal femur fracture: can double plating via a modified Olerud extensile approach be a standby solution?. J Orthop Traumatol Off J Italian Soc Orthop Traumatol.

[32] Ziran BH, Rohde RH, Wharton AR (2002). Lateral and anterior plating of intra-articular distal femoral fractures treated via an anterior approach. Int Orthop.

[33] Metwaly RG, Zakaria ZM (2018). Single-incision double-plating approach in the management of isolated, closed osteoporotic distal femoral fractures. Geriatr Orthop Surg Rehabil.

[34] Steinberg EL, Elis J, Steinberg Y, Salai M, Ben-Tov T (2017). A double-plating approach to distal femur fracture: a clinical study. Injury.

[35] Çiçek H, Tuhanioğlu Ü, Oğur HU, Seyfettinoğlu F, Bozkurt M (2018). An alternative treatment for osteoporotic Su Type III periprosthetic supracondylar femur fractures: double locking plate fixation. Acta orthopaedica et traumatologica turcica.

[36] Park K-H, Oh C-W, Park K-C, Kim J-W, Oh J-K, Kyung H-S, Kim H-J, Yoon Y-C (2020) Excellent outcomes after double-locked plating in very low periprosthetic distal femoral fractures. Arch Orthop Trauma Surg 10.1007/s00402-020-03655-5 33128096

[37] Bologna MG, Claudio MG, Shields KJ, Katz C, Salopek T, Westrick ER (2020). Dual plate fixation results in improved union rates in comminuted distal femur fractures compared to single plate fixation. J Orthop.

[38] Su ET, DeWal H, Di Cesare PE (2004). Periprosthetic femoral fractures above total knee replacements. Am Acad Orthop Surg.

[39] Chapman MW, Finkemeier CG (1999). Treatment of supracondylar nonunions of the femur with plate fixation and bone graft. J Bone Jt Surg Am Vol.

[40] Laflamme GY, Leduc S, Petit Y (2012). Reattachment of complex femoral greater trochanteric nonunions with dual locking plates. J Arthroplast.

[41] Peng Y, Ji X, Zhang L, Tang P (2016). Double locking plate fixation for femoral shaft nonunion. Eur J Orthop Surg Traumatol Orthop Traumatol.

[42] Nagy MT, Pydisetty G, Kwaees TA, Saldanha K (2020) Outcome of revision surgery for bisphosphonate related subtrochanteric fracture non-union following failed intramedullary nailing. Injury 10.1016/j.injury.2020.09.051 33092855

[43] Lu J, Guo S-C, Wang Q-Y, Sheng J-G, Tao S-C (2020). J-bone graft with double locking plate: a symphony of mechanics and biology for atrophic distal femoral non-union with bone defect. J Orthop Surg Res.

[44] Mardani-Kivi M, Karimi Mobarakeh M, Keyhani S, Azari Z (2020). Double-plate fixation together with bridging bone grafting in nonunion of femoral supracondylar, subtrochanteric, and shaft fractures is an effective technique. Musculoskelet Surg.

[45] Sun L, Li Z, Ma T, Xue H-Z, Wang Q, Lu D-G, Lu Y, Ren C, Li M, Zhang K (2019). Treatment of atrophic nonunion via autogenous ilium grafting assisted by vertical fixation of double plates: a case series of patients. J Int Med Res.

[46] Alonso J, Geissler W, Hughes JL (1989). External fixation of femoral fractures. Indications and limitations. Clin Orthop Relat Res.

[47] Geissler WB, Powell TE, Blickenstaff KR, Savoie FH (1995). Compression plating of acute femoral shaft fractures. Orthopedics.

[48] Rüedi TP, Lüscher JN (1979). Results after internal fixation of comminuted fractures of the femoral shaft with DC plates. Clin Orthop Relat Res.

[49] Riemer BL, Butterfield SL, Burke CJ, Mathews D (1992). Immediate plate fixation of highly comminuted femoral diaphyseal fractures in blunt polytrauma patients. Orthopedics.

[50] Ebraheim NA, Martin A, Sochacki KR, Liu J (2013). Nonunion of distal femoral fractures: a systematic review. Orthop Surg.

[51] Chan DB, Jeffcoat DM, Lorich DG, Helfet DL (2010). Nonunions around the knee joint. Int Orthop.

[52] Moore TJ, Watson T, Green SA, Garland DE, Chandler RW (1987). Complications of surgically treated supracondylar fractures of the femur. J Trauma.

[53] Barrack RL, Butler RA (2005). Current status of trochanteric reattachment in complex total hip arthroplasty. Clin Orthop Relat Res.

[54] Takahira N, Itoman M, Uchiyama K, Takasaki S, Fukushima K (2010). Reattachment of the greater trochanter in total hip arthroplasty: the pin-sleeve system compared with the Dall-Miles cable grip system. Int Orthop.

[55] Koyama K, Higuchi F, Kubo M, Okawa T, Inoue A (2001). Reattachment of the greater trochanter using the Dall-Miles cable grip system in revision hip arthroplasty. J Orthop Sci Off J Japn Orthop Assoc.

[56] Ritter MA, Eizember LE, Keating EM, Faris PM (1991). Trochanteric fixation by cable grip in hip replacement. J Bone Jt Surg Br Vol.

[57] Hamadouche M, Zniber B, Dumaine V, Kerboull M, Courpied JP (2003). Reattachment of the ununited greater trochanter following total hip arthroplasty. The use of a trochanteric claw plate. J Bone Jt Surg Am Vol.

